# Supplementary material for: Seroprevalence of hospital staff in a province with zero COVID-19 cases
Source: PLoS One. 2021 Apr 1;16(4):e0238088. doi: 10.1371/journal.pone.0238088 (PMC8016267; doi:10.1371/journal.pone.0238088)
Supplement: Attachment — Submitted filename: Response to Reviewer Ranong.docx [file pone.0238088.s002.docx]

Dear Editor,

We thank you and the reviewers for the comments and suggestions. Please find our point-by-point responses below:

Editor: Thank you for submitting your manuscript to PLOS ONE. After careful consideration, we feel that it has merit but does not fully meet PLOS ONE’s publication criteria as it currently stands. The data presented is very limited (only a one month period). More recent surveillance of the same cohort will be preferable and compared to the results obtained from the earlier time point. Therefore, we invite you to submit a revised version of the manuscript that addresses all the points raised during the review process.

Response: Thank you for your time and consideration. The manuscript was revised to meet PLOS ONE’s publication criteria. While the data is relatively limited, this data is the best evidence we can obtain in the current situation in Thailand where the appropriate COVID-19 test in population has not been conducted. Moreover, as the hospital underwent a major significant change in its organizational structure and management team, along with the compliance to the ethical approval we initially received, our request to conduct repeat surveillance of the same cohort has not been granted yet and might take several months. Nonetheless, please note that, as of December 7, 2020, this province still reports zero cases. We added these limitation statements to the Discussion section accordingly.

Editor: 2.We note that [Figure(s) 1] in your submission contain map images which may be copyrighted. All PLOS content is published under the Creative Commons Attribution License (CC BY 4.0), which means that the manuscript, images, and Supporting Information files will be freely available online, and any third party is permitted to access, download, copy, distribute, and use these materials in any way, even commercially, with proper attribution. For these reasons, we cannot publish previously copyrighted maps or satellite images created using proprietary data, such as Google software (Google Maps, Street View, and Earth).

Response: As Figure 1 contains original data from our research, we provide a replacement figure with an original image from our colleague whom we acknowledged in the revised manuscript so the copyright issue should no longer be a concern. As of December 7, 2020, this province still reports zero cases so we updated the data in Figure 1 as well.

Editor: 3.Thank you for stating the following in the Acknowledgments Section of your manuscript:

[We thank Baiya Phytopharm, Thailand for supporting the Baiya Rapid COVID-19 IgG/IgM test kit. The company did not involve in the data analysis, interpretation, or manuscript preparation.]

We note that you have provided funding information that is not currently declared in your Funding Statement. However, funding information should not appear in the Acknowledgments section or other areas of your manuscript. We will only publish funding information present in the Funding Statement section of the online submission form.

Please remove any funding-related text from the manuscript and let us know how you would like to update your Funding Statement. Currently, your Funding Statement reads as follows:

[The author(s) received no specific funding for this work.]

Additionally, because some of your funding information pertains to [commercial funding//patents], we ask you to provide an updated Competing Interests statement, declaring all sources of commercial funding.

Response: We did not mean to provide funding information in the Acknowledgment Section. None of the authors receive any funding from the company mentioned in the Acknowledgment Section. We only wanted to thank them for their contribution to developing the local COVID-19 IgG/IgM test kit for Thailand upon request free of charge, which resulted in an opportunity for us to conduct this observational study. We revised the choice of words in the Acknowledgment Section accordingly.

Editor: In your Competing Interests statement, please confirm that your commercial funding does not alter your adherence to PLOS ONE Editorial policies and criteria by including the following statement: "This does not alter our adherence to PLOS ONE policies on sharing data and materials.” as detailed online in our guide for authors http://journals.plos.org/plosone/s/competing-interests. If this statement is not true and your adherence to PLOS policies on sharing data and materials is altered, please explain how. Please include the updated Competing Interests Statement and Funding Statement in your cover letter. We will change the online submission form on your behalf.

Response: Authors have no competing interests according to the PLOS definition. The suggested statement is added in the Competing Interests statement. The updated Competing Interests Statement and Funding Statement were included in the cover letter.

Editor: 4.We noticed you have some minor occurrence of overlapping text with the following previous publication(s), which needs to be addressed: https://www.medrxiv.org/content/10.1101/2020.06.24.20139188v1?versioned=true

In your revision ensure you cite all your sources (including your own works), and quote or rephrase any duplicated text outside the methods section. Further consideration is dependent on these concerns being addressed.

[Note: HTML markup is below. Please do not edit.]

Response: All sources used in this manuscript are already cited in the previously submitted manuscript (including the concerning publication mentioned above – Ref. 12). All overlapping text outside the methods section is rephrased. Please advise if we still have to address this concern differently.

Reviewer #1: This manuscript by Nopsopon et al described the SARS-CoV-2 seroprevalence of the hospital staff of one of the largest hospitals in the Ranong province of Thailand, where no confirmed COVID-19 was found by between April-May, 2020. The testing was carried out using a homemade IgM/IgG detection test. The authors found that among the 844 members tested only 7 cases were found to be positive for IgM but negative for IgG. Only 1 of the positive cases has reported sore throat. All the positive cases were PCR negative. This is the first report of the testing of hospital staff in the region, however, based on numerous reports it is hardly surprising that there are individuals who have prior exposure to the virus but have yet remained asymptomatic. The data presented is very limited and for only a one month period, way back in April-May. With the fast changing pandemic more recent surveillance of the same cohort will be preferable and compared to the results obtained from the earlier time point. The addition data will substantially strengthen the manuscript. In addition, there are several other concerns.

Response: Thank you for your time in reviewing the manuscript. Despite the limitation of data in this manuscript, we provide the best evidence available in the current situation in Thailand where COVID-19 testing is low, given the limited resources and national screening policy. With the volatile nature of the pandemic, we would like to provide evidence of an actual situation where the zero or low number of COVID-19 confirmed cases in Thailand might come from a low number of people tested. Although we wanted to conduct a repeat survey as suggested, the hospital underwent a major significant change in its organizational structure and management team, along with the compliance to the ethical approval we received; hence, our request to conduct repeat surveillance of the same cohort has not been granted and might take several months. We added these limitation statements to the Discussion section accordingly (Line 194-198). We believe that the data we provided would bring attention to the overlooked problem of the low number of testing in this region, thus encourage the governing body to take more action and change the perspective that only high-risk or symptomatic patients should get tested for mass screening to get the actual situation in Thailand.

Reviewer #1: 1. It is not clear why only IgM and not IgG was detected. This is quite surprising. This needs to be followed up and explain whether this could be due to experimental errors. In addition, were the cases being followed up and retested for Ab and for IgG?

Response: More discussion on predominant IgM positive compared to IgG positive is provided in Discussion section. Mass screening or antibody testing has not been readily supported in Thailand, especially in the current situation where the national policy allows people to get tested as low as possible to keep the low number of confirmed cases. We really would like to conduct a follow-up test to provide the most recent situation in Thailand; however, without the first piece of evidence to demonstrate how mass screening would provide benefit and reality of COVID-19 situation in Thailand, the follow-up test is currently not possible. The cases were not retested for Antibody but instead get PCR testing which, at that time, was considered the gold standard for COVID-19 confirmation in Thailand.

Reviewer #1: 2. There is a concern on the test sensitivity of 94.1% (48 of 51), some positive cases could have been missed.

Response: We concern about some positive cases would be missed by this rapid IgM/IgG test kit we used; however, this is the best readily available test that could be used as mass screening in Thailand at the time we conducted the study. As of December 7, 2020, this province still reports zero cases.

Reviewer #1: 3. Was second Ab tested carried out with the IgM positive cases?

Response: The cases were not retested for Antibody but instead get PCR testing which, at that time, was considered the gold standard for COVID-19 confirmation in Thailand.

Reviewer #1: 4. It is not clear whether any hospital staff were excluded. It was indicated in line 106 that “Participants with active symptoms suiting national criteria for polymerase chain reaction testing were quarantined and excluded.

Response: We are sorry for the confusion and thank you very much for pointing that out. The statement “Participants with active symptoms suiting national criteria for polymerase chain reaction testing were quarantined and excluded.” was in the Methods section to describe how our observational study concurs with the current COVID-19 screening policy in Thailand. Hence, no staff was excluded from our study per se.

Reviewer #1: 5. It is not clear whether 82 or 100 individuals were tested twice, conflicting numbers were provided in the abstract versus the text.

Response: Again, we are sorry for the confusion. We revised both the “Repeating antibody tests in random sampling participants” subsection of the Method section as follows: “Of 844 participants, 100 were randomly selected to get repeating antibody testing in April and May. None of them had any immunoglobulin developed in April. Of 100 randomly selected participants, 82 participated in antibody testing in May (response rate for repeating antibody test 82%) which showed no antibody against COVID-19.” The Abstract has also been revised accordingly.

Reviewer #2: Burden of disease deals with a range of medical statistics aimed to give a comprehensive picture of how diseases impact on society or to allocate health care and research resources. Diagnostic tests are an important part in this regard. A perfect test, usually referred to as the gold standard implies being positive in all patients with the disease and negative in all patients who do not have the disease. However, most tests are imperfect. In the work by Pongpirul et al. the authors carried out a survey assessing the presence of anti-COVID-19 antibodies in hospital workers from a Thailand area in which COVID-19 had not been reported during the study period. Among 844 participants few of them (<1%) revealed the presence of IgM antibodies bearing no close relationship with their clinical or personal characteristics. The study adds some information to the local features of COVID-19 infection.

Response: Thank you very much. We are pleased to know that our study is useful.

Reviewer #2: It would be interesting to know whether there existed a different degree of exposure risk among staff members.

Response: Staff members consist of many occupations which have various degree of exposure risk, thus we provide the history of close contact confirmed COVID-19 case and close contact suspected COVID-19 case to assess association with a history of risk exposure and seroprevalence.

Reviewer #2: It is intriguing that people yielding an IgM positive test at the beginning failed to develop IgG response a month later. Any tentative explanation for this?

Response: Staffs with IgM positive did not have an antibody test a month later, instead they have PCR testing which was considered the gold standard at that time. For repeating antibody testing, we randomly selected 100 from 844 staff in Ranong hospital to get antibody testing twice in April and May, which none of them developed antibodies against SARS-CoV-2.

Reviewer #2: Information is needed on whether validation studies were carried in recent COVID-19 cases, or not?

Response: Yes, the validation study for the rapid test kit was conducted in recent COVID-19 cases.

Reviewer #2: In discussing results please note that lack of association may depend on the reduced number of people yielding a positive IgM response.

Response: Thank you very much. The discussion about lack of association may depend on the reduced number of people yielding a positive IgM response is provided in the Discussion section as advised (Line 174-175).

Reviewer #2: Page 8, lines 191-192, there are more studies in this regard which should be mentioned.

Response: Additional studies are added as suggested (Line 182-193).

Reviewer #2: Please also note that the time when Ab are detected may have nothing to do with transmission.

Response: The discussion about the time when Ab is detected may have nothing to do with the transmission is provided in the Discussion Section.

Reviewer #2: The Bangkok Metropolitan area might be indicated in the Figure.

Response: The Bangkok Metropolitan area is now indicated in the revised Figure.

We hope that our responses are satisfactory. Should there be anything that might improve our work, please kindly inform us. Thank you very much for your kind consideration.

Best Regards,

Assist. Prof. Dr. Krit Pongpirul, MD, MPH, PhD.

On behalf of the authors
