# Supplementary material for: Seroprevalence of hospital staff in a province with zero COVID-19 cases
Source: PLoS One. 2021 Apr 1;16(4):e0238088. doi: 10.1371/journal.pone.0238088 (PMC8016267; doi:10.1371/journal.pone.0238088)
Supplement: Attachment — Submitted filename: Response to Reviewer Ranong 2.11 TN KP clean.docx [file pone.0238088.s003.docx]

Dear Editor,

We thank you and the reviewers for the comments and suggestions. Please find our point-by-point responses below:

Editor: Thank you for submitting your manuscript to PLOS ONE. After careful consideration, we feel that it has merit but does not fully meet PLOS ONE’s publication criteria as it currently stands. Therefore, we invite you to submit a revised version of the manuscript that addresses all the points raised during the review process.

Response: Thank you for your time and consideration. The manuscript was revised to meet PLOS ONE’s publication criteria. The concerned limitation was discussed in detail in the Discussion section. The result interpretation, wording choices, and typos were revised throughout the manuscript.

Reviewer #1: The authors has addressed the concerns as best as they could without performing the requested additional testing results, which this reviewer still think is not satisfactory. Perhaps adding the rationale and explanation for having IgM and no IgG response in the tested case will provide additional scientific interest for the readers.

Response: Thank you for your suggestion. The rationale and explanation for having IgM and no IgG response in the tested case were added in the Discussion section.

Reviewer #3: 1) Although I agree with the flaws/limitations of the study as summarized by Reviewer 1, the study does add informative descriptive data for this region in Thailand despite the limitations.

Response: Thank you for your supportive comments. We believe that this study provides a useful piece of evidence for COVID-19 pandemic control from the region with a unique situation.

Reviewer #3: 2) The statement that females have higher IgM % than males in the Abstract and Results sections is not appropriate. Just provide the percentages and confidence intervals (CIs). As provided in the Results section, the CIs are nearly overlapping so it cannot be concluded that they are different. Please revise accordingly the statements below.

Abs: Females seemed to have higher IgM seropositive than male staff (1.0% vs. 0.5%).

Results: Female staff seemed to have higher rate of positive IgM (1.0%, 95% CI: 0.5–2.1%) than male (0.5%, 95% CI: 0.1–2.6%).

Response: Both statements were revised accordingly by providing only the percentages and confidence intervals (CIs) of each gender.

Reviewer #3: 3) In the conclusion: Consider revising FROM:

“Antibody testing should be promoted for mass screening, particularly in asymptomatic healthcare workers.”

TO:

“Accurate antibody testing is a valuable screening tool, particularly in asymptomatic healthcare workers.”

Response: The statement in the conclusion was revised accordingly.

Reviewer #3: 4) Typo:

Page 7: Only 1% of participants had previous negative PCR results while the rest never get tested (Table 1). … should be ‘got’ tested. Please read the paper again carefully and correct any grammatical errors.
Response: The typo on Page 7 was corrected. The manuscript was perused by the authors for any typo or grammatical error which was corrected accordingly.

We hope that our responses are satisfactory. Should there be anything that might improve our work, please kindly inform us. Thank you very much for your kind consideration.

Best Regards,

Assoc. Prof. Dr. Krit Pongpirul, MD, MPH, PhD.

On behalf of the authors
